# Tuberculosis: Just the FAQs

**DOI:** 10.1128/mBio.01910-17

**Published:** 2017-12-19

**Authors:** Kristine M. Guinn, Eric J. Rubin

**Affiliations:** Department of Immunology and Infectious Diseases, Harvard T. H. Chan School of Public Health, Boston, Massachusetts, USA; New York University School of Medicine

**Keywords:** infectious disease, public health, tuberculosis

## Abstract

Tuberculosis is responsible for more deaths worldwide than any other infectious disease. For anyone looking to learn more about this persistent public health threat, this conversational “frequently asked questions” style review addresses a breadth of questions. It offers a brief, somewhat opinionated, review of what is and is not known, particularly in light of how findings in the lab do or do not help inform the understanding of human tuberculosis.

## PERSPECTIVE

Several reviews have been published recently that cover tuberculosis (TB) in both broad and specific terms. Many of these reviews are authoritative and cover various areas in depth. But they are not easy to read on your phone. In an effort to address that gap, here we present all of TB in a handy frequently asked question (FAQ) format. This required us to condense a good deal of information, so we have done so through the lens of our own biases. So please read on, but *caveat lector*!

Importantly, TB is a disease of humans. While there is a considerable body of *in vitro* research, the causative organism, *Mycobacterium tuberculosis*, has no environmental niche and does not naturally infect animals. Thus, in nature, it is essentially an obligate human pathogen. Any experimental system outside humans is, by definition, a model. And models rise and fall on how well they mimic and predict what happens under actual conditions. Thus, here we will examine what we know about TB in the context of human infection and disease. This will exclude a vast body of interesting and potentially important research, but it provides a framing device to understand what we know and, more importantly, what we don’t know.

**How much TB is out there?**

*It depends on how you count*.

TB is a common disease. Because international standards require that all cases be reported to health authorities, we should have an accurate measure of the total burden of disease. But we don’t. Reporting is incomplete, often due to the lack of reporting infrastructure in the places where TB is endemic. Moreover, TB is difficult to diagnose, and many cases go unidentified and uncounted. Thus, we must rely on modeling approaches to take the reported data that exist and estimate the total actual number of cases. The best estimates probably come from two organizations, the World Health Organization (1), which has a dedicated TB division, and the International Health Metrics and Evaluation (IHME) Global Burden of Disease project. The most recent WHO report puts the number of incident cases in 2015 at 10.4 million (1), while the IHME estimates 8.8 million (2) (http://vizhub.healthdata.org/gbd-compare/). The estimated numbers of deaths attributable to TB during the same year were 1.4 and 1.1 million by the WHO and IHME, respectively.

Neither of these numbers sounds good. And they are not. According to IHME statistics, for the first time in many years, TB killed more people than human immunodeficiency virus (HIV), the previous leading infectious disease killer of adults. Now TB has taken that dubious crown.

**What is the disease?**

*Mostly pneumonia*. *But almost any organ can be infected*.

Classically, we think of pneumonia as an infection of the airways and alveolar spaces with the consequent influx of inflammatory cells and edema fluid. But the pneumonia caused by *M. tuberculosis* is different, with impairment of the lung tissue itself rather than the airways. TB is characterized by a particular hallmark, the granuloma. This is a collection of immune and inflammatory cells with a distinctive rounded architecture, generally with macrophages in the center surrounded by a ring of lymphocytes. The granuloma can be found in many conditions that induce chronic localized inflammation, including histoplasmosis, tularemia, and leishmaniasis. However, several features are moderately distinctive for TB—an area of central cell death (producing “necrotizing” granulomas), lipid-filled “foamy” macrophages and “giant cells,” multinucleate syncytia formed from fused macrophages. As necrosis proceeds, the boundaries of the granuloma can erode into airways, allowing their contents to be aerosolized (and releasing transmissible bacteria into the environment) and resulting in cavities with loss of lung tissue. Not all types of lesions exist in all patients, but generally, those with cavitary TB have the most severe pulmonary disease. A less common type of disease, miliary TB (so-called because the chest radiograph appearance looked like millet seeds to early radiologists), results in widespread infection throughout the body without granuloma formation. This is most often seen in the setting of immune compromise.

Classically, there was thought to be a strict progression from inflammatory lesions to granulomas with subsequent necrosis and cavity formation. However, recent evidence suggests that this is an oversimplification. Animal models have shown that granulomas are far more dynamic, progressing and regressing in single individuals (3). Human data are scarce, but serial imaging of patients undergoing antibiotic therapy and monitored using positron emission tomography coupled with computed tomography (PET/CT), a method that allows simultaneous three-dimensional visualization of the degree of inflammation and high-resolution tissue structure, has shown that individual lesions have different fates (4–6), something also seen in nonhuman primates (NHPs) with or without therapy (5, 7–10). This suggests a newer concept—each granuloma is an individual battlefield where the balance between the pathogen and host defense is, to some degree, sequestered from the larger war. Who benefits from this sequestration is unclear—is the organism protected against a global assault, or does the granuloma limit the spread of infection?

TB is notoriously stealthy. The preceding description is of “active TB” disease. But the majority of people who acquire aerosol infection go straight to “latent TB,” with no clinical symptoms. This is described in the discussion of “Who doesn’t get sick?”

**Who gets sick?**

*Specific high-risk groups. And many others as well*.

The incidence of TB worldwide had been falling slowly for many years until the onset of the HIV epidemic. That was the spark that fueled a large resurgence in TB. The areas of the world where HIV is most common are also the areas where TB is endemic. This unfortunate colocalization resulted in TB being the single leading cause of death among HIV-infected individuals (11).

Why are HIV patients at such an increased risk of developing illness once infected with TB? The answer is not simple. Certainly, as discussed below, CD4^+^ T lymphocytes play a vital role in controlling TB infection. HIV patients with very low CD4^+^ T cells are particularly vulnerable to TB. Interestingly, patients with advanced HIV disease tend to develop disseminated TB and other disease that is not localized to the lung, so-called extrapulmonary TB. This suggests that the lung represents a unique immunologic environment where *M. tuberculosis* has some specific advantage, and that advantage is lost in HIV.

However, it is not all down to CD4^+^ cells (11). Even with high numbers of CD4^+^ cells, early after infection, HIV patients are at increased risk of TB, though that disease is largely confined to the lung. The mechanism for this increased risk is unknown.

Diabetes mellitus patients also develop TB at higher rates than nondiabetic patients, though the risk is much lower than for HIV. However, since diabetes is so common, it probably represents the single largest risk factor for TB worldwide (12).

Others are at increased risk also. Nutritional deficiencies appear to predispose to infection, and strikingly, those who have their stomach removed (gastrectomy) or who have malabsorption of multiple nutrients are at particular risk of TB (13). A number of studies have looked for genetic determinants that increase susceptibility to TB. Overall, these studies have been disappointing—many of the genetic determinants are not replicated in independent populations, and almost all produce small effects. Notable exceptions are defects in the gamma interferon (IFN-γ)–interleukin 12 (IL-12)–IL-23 signaling pathway. Several rare alleles that result in increased TB susceptibility have been described (14). This susceptibility is so extreme that many of these individuals succumb to infections with more commonly encountered (but less virulent) environmental mycobacteria before they are ever exposed to *M. tuberculosis*.

**Who doesn’t get sick?**

*Everyone else. Even after they get infected*.

In fact, developing disease after becoming infected with *M. tuberculosis* is the exception, not the rule. We know this because we have crude measures of infection that are independent of symptoms. Many individuals who are exposed to someone with TB in turn develop a specific immune response to *M. tuberculosis*, measured either by a delayed type hypersensitive response to crude antigens (the tuberculin skin test) or by IFN-γ release from whole blood cells stimulated with purified *M. tuberculosis* antigens (interferon gamma release assays [IGRAs]). Since we know that these individuals are at risk for developing disease later, we assume that they are infected. Using these measures, it appears that the vast majority of people do not develop symptoms following infection, a condition known as latency. However, 5 to 15% of those that are latently infected will eventually develop active disease, some as long as decades after the initial infection (15, 16). Thus, in many individuals, bacteria can clearly persist for extended times.

**What is the biological basis of latency?**

*Don’t know. In fact*, *we’re not really sure what it is*.

Latency is a distinctive feature of TB. Of those that do get sick, a minority develop symptoms shortly after exposure. Others become ill months to years after they become infected. In fact, latency is by far the most common outcome of infection.

What is happening during that time? For many years, the debate has revolved around the bacteria—are they actively growing, or have they entered a nonreplicating state? Much of the research in this area has centered on the latter hypothesis, that during latency *M. tuberculosis* persists without growing or causing a pathological response. Experiments in animal models have been inconclusive, with some suggesting ongoing balanced growth and death while others imply a lack of growth (7, 17). And this is all complicated by the possibility that many organisms might adopt a physiological state where they are difficult to culture *in vitro* (18–20).

The major limitation of all of these observations is that they are largely made in animals in which latency does not occur without considerable experimental manipulation. Thus, we do not know how much can be extrapolated to human infection. One recent approach has been to study this phenomenon in NHPs, which have disease that more closely replicates human TB (21). Upon infection with low doses of bacteria, about half of NHPs develop progressive infection, while the other half remain clinically well, at least by the measures that can be easily evaluated. Upon examination, the “well” animals actually have a spectrum of subacute infection, ranging from low to high bacterial load and concomitant inflammation. This suggests a very different model—that all of latency represents ongoing infection, with individuals with the highest bacterial load at the highest risk of developing TB disease (22).

Which of these models reflects human disease? We are now gaining some insights by monitoring asymptomatic HIV patients with positive IGRA tests, a population at high risk of disease, using PET/CT imaging (23). Those with evidence of subclinical disease were more likely to progress to active infection. Ongoing trials are using the same imaging modality to evaluate household contacts of TB patients. While PET/CT is a very indirect measure of infection, it is a sensitive assay for the tissue reaction to bacteria. These natural history studies could help establish the importance of the concept of latency as a spectrum of subacute infection.

**How is TB transmitted?**

*From your family and friends. Or strangers*.

Under ordinary circumstances, TB can be transmitted only via aerosol. Thus, it requires a source patient with active TB who is coughing up microdroplets that contain *M. tuberculosis* and sufficiently close and prolonged contact to allow someone else to inhale those microdroplets. Transmission usually occurs between family members and close contacts, due to frequency and duration of exposure. It can also occur between coworkers who work in close proximity (as is seen in the gold mines of South Africa [24]) and, less often, between strangers (as has been observed on public transportation [25]).

Considerable evidence and common sense suggest that the more bacteria in the aerosol, the more infectious the source patient. This leads to an interesting corollary. Since TB is not generally a disease of the airways, the most efficient method of transmission is through breakdown of granulomas into airways. Thus, aside from rare upper airway infection, those with cavitary TB are the most efficient transmitters (26, 27). From the standpoint of teleology, *M. tuberculosis* benefits from the formation of cavities because this pathology allows for the most efficient spread. Conversely, those with miliary TB have very high organism burdens but rarely spread, as the bacteria have little access to the airways.

**How is TB diagnosed?**

*Largely by methods devised more than a century ago. But that is changing*.

While the methods devised by Robert Koch in the 1880s for visualizing and culturing *M. tuberculosis* have been improved upon, the basic concept has remained the same. In most of the world, diagnosis relies on sputum smear microscopy using a dye specific for the mycobacterial cell wall as a probe for the bacilli. This is fairly insensitive and probably misses as many as half of the cases of pulmonary TB (and pretty much all extrapulmonary TB cases). Sputum culture is much better, with a sensitivity estimated at ~90%, but culture is both difficult and slow, requiring specialized facilities and several weeks of incubation. More modern approaches that rely on cultivation in broth instead of solid media and use automatic detection methods are more rapid but require specialized equipment and are not available in many areas where TB is endemic (28).

Clearly, DNA amplification would be more rapid. Many “home brew” approaches had uneven sensitivity, probably because it is difficult to release DNA from mycobacteria with their thick and waxy cell walls. Recently, however, this process has been automated in a cartridge-based system that requires little human intervention (29). The resultant commercial system has an important additional bonus—using molecular beacons, it can detect polymorphisms responsible for resistance to rifampin, one of the key drugs used in treating TB. The rapid diagnosis and determination of drug susceptibility are potentially transformative, as it enables effective therapy to begin immediately.

**How is TB treated?**

*With lots and lots of drugs*.

A series of clinical trials performed in India finally arrived at today’s TB treatment regimen—four drugs are administered for 2 months after which two are dropped and the remaining two are continued for an additional 4 months (30).

Why so many drugs? There are two rationales. First, as seen shortly after the introduction of the first antituberculous drug, streptomycin, resistance arises rapidly from treatment with less-complex regimens. And, second, this combination of drugs allows shortening treatment, reducing the length of therapy from more than a year to 6 months.

Of course, this “short-course” treatment hardly sounds very impressive compared to most other antibiotic therapy. The current regimen was arrived at purely empirically—by trial and error (31, 32). The underlying biology of the slow response to treatment is still unclear. Most current studies focus on inherent properties of the bacteria—their ability to enter nonreplicating states and their ability to display drug tolerance (the ability to generate subpopulations of cells that are not genetically resistant but are more slowly killed by antibiotics) (33–35). Recent work, however, suggests that many of the drugs used to treat TB might not penetrate effectively into some granulomas (36). The contribution of each of these factors remains unknown.

**How is TB prevented?**

*By staying away from coughing people. And by treating the coughers*.

All effective TB control measures rely on separating people from viable airborne *M. tuberculosis*. These control measures range from low tech (such as quarantine and open windows to increase ventilation on TB wards) to high tech (high-efficiency face masks and UV irradiation of room air). The most effective means of control, however, is probably treatment. Upon starting therapy, infected individuals rapidly become much less likely to transmit disease even though cure requires a far longer time. In fact, treatment is the mainstay of TB control in areas where disease is highly endemic, as it is impractical to quarantine patients with active disease.

A vaccine that could prevent TB could represent the ultimate control measure. In fact, a vaccine is in use widely throughout the world. *Mycobacterium bovis* BCG, a live attenuated strain developed in the 1930s at the Pasteur Institute, is administered to infants in most countries where TB is prevalent. This vaccine clearly decreases the incidence of childhood TB, a disease associated with dissemination and high rates of morbidity and mortality. Unfortunately, it is far less effective against adult TB; in fact, the largest single study showed no efficacy whatsoever in an area where TB is endemic (37). Thus, it does not represent an effective tool for controlling the TB epidemic, as is borne out by continued high TB incidence despite many decades of BCG use in countries where TB is prevalent.

**How did *M. tuberculosis* evolve?**

*From slime. Like the rest of us*.

Though they defy the rules of both Gram-positive and Gram-negative categories, mycobacteria are phylogenetically Gram-positive bacteria that belong to the rather diverse group of organisms with high G+C content in their genomes. This puts them among bacteria that are quite distantly related to the most heavily studied model organisms, *Escherichia coli* (a Gram-negative bacterium) and *Bacillus subtilis* (a low-G+C Gram-positive bacterium). Thus, much of the metabolism of these organisms is dissimilar from either of the common bacterial models. Most strikingly, mycobacteria have a complex cell wall composed of multiple covalently and noncovalently linked layers. While many of the components are known, the actual ultrastructure is not known. Moreover, ~70% of mycobacteria (dry weight) is composed of lipids, including many that are not made by any other bacteria. Much of the metabolic machinery is devoted to lipid biosynthesis and degradation, and several lipids play key roles in pathogenesis.

Most mycobacteria are environmental organisms that are found in water and soil. Habitats such as peat bogs are particularly rich sources of mycobacteria. Although most are not important human pathogens, many can infect other hosts as diverse as frogs and birds. And most mycobacteria share the ability to survive inside environmental phagocytes such as amoebas (38, 39). Thus, the capacity of *M. tuberculosis* to survive within macrophages might be derived from its ancestors.

**How diverse is *M. tuberculosis*?**

*Not very. But perhaps enough to have a clinically significant impact*.

It comes down to whether you are a splitter or a joiner. *M. tuberculosis* strains belong to several subspecies that make up a complex. But, for the most part, these are divisions that only a taxonomist can love, as all of the members are quite similar to one another. Compared to most other pathogenic bacteria, *M. tuberculosis* has very limited genetic diversity.

But what diversity does exist has one important practical implication and might give us clues to the evolution of the pathogen. No horizontal gene transfer in *M. tuberculosis* has been detected, so all changes occur through mutation. Mutations occur slowly over time and seem relatively independent of the growth rate (40). Thus, allelic variation can be used as a marker of strain ancestry and degree of relatedness. And different genetic clocks run at different rates with both rapid (produced by insertion sequences and genetic repeats) and slow (single-nucleotide substitutions) diversification producing different sorts of markers. This allows investigators to monitor single outbreaks through a population or to determine the long-term association of strains with populations (41, 42). The latter work has suggested an interesting hypothesis—specific strains have adapted to specific human populations (43). While it seems likely that *M. tuberculosis* has coevolved with human subgroups, it is less clear whether this represents specific adaptations or genetic drift.

**How does *M. tuberculosis* become drug resistant?**

*Independently. They don’t like to share*.

As *M. tuberculosis* is unable to acquire DNA from other bacteria, all resistance must arise anew within strains. Resistant strains can then be transmitted between people, but resistance genes are not swapped among strains. The rate of resistance to any given antibiotic ranges from ~10^−6^ to 10^−9^, largely dictated by the size of the potential target for mutagenesis. Drugs that require activation by cellular processes, (e.g., isoniazid by the product of the *katG* gene) have relatively high rates, while those where mutations must be in localized regions of a specific target gene (e.g., *rpoB*, the target of rifampin) occur much more rarely (44). Since organism burdens can be quite high during infection, each of these rates is high enough so that, if treated with a single drug, resistance develops reproducibly. And, since the vast majority of mutations mediate resistance only to a single drug class or a single target, there are no true multidrug resistance mutations.

Since standard treatment involves multiple drugs, resistance rarely develops during therapy. However, there is still quite a bit of multidrug resistance among circulating strains, perhaps as many as 500,000 cases per year (1). Resistant strains are already present in the population, and even those that are highly resistant are fully capable of spreading widely through the population. In fact, the spread of these resistant organisms, rather than newly arising resistance, accounts for most of the burden of highly resistant disease (45).

**What do model pathogens tell us about TB?**

*It depends on the question*.

**(i) *M. tuberculosis*.** Clearly, the best model is no model at all. Genetic methods have improved markedly, now allowing transposon mutagenesis, site-specific integration, and various efficient methods of allelic replacement in *M. tuberculosis* (46–48). And, using the imaging modalities that are available for humans, it is possible to monitor the course of disease in some living animals (49–52). But *M. tuberculosis* is still quite difficult to work with, for two major reasons: it grows very slowly, taking weeks to form a colony on solid medium, and it requires a biosafety level 3 (BSL-3) facility. Thus, model organisms still play a critical role.

**(ii) *M. bovis* BCG.** The *M. bovis* BCG vaccine strain has been administered to millions of infants who lack mature immunity with little evidence of pathology. This remarkable safety record means that this organism can be handled outside the BSL-3 lab, in a drastically more convenient BSL-2 lab. Unfortunately, this organism grows as slowly as *M. tuberculosis* does. And it is a poor pathogen—able to persist but not grow or cause disease in many animal models. Moreover, there are key physiological differences between the parental species, *Mycobacterium bovis* and *M. tuberculosis*. For example, *M. bovis* strains lack *pncA*, the enzyme necessary to convert the prodrug pyrazinamide to its active form (53).

**(iii) Attenuated *M. tuberculosis*.** Several strains of *M. tuberculosis* have been either naturally selected or genetically engineered for attenuation. *M. tuberculosis* H37Ra has many genomic changes compared to wild-type bacteria. The basis for its attenuation is due, in part, to loss of the normal activity of a key transcriptional regulator, PhoP (54). In addition, H37Ra has many other single-nucleotide polymorphisms relative to its parental strain, most of uncertain significance (55).

There are also several strains that have been deliberately constructed to lack multiple growth or virulence determinants, including the ability to synthesize key nutrients or to produce key secreted proteins (56, 57). These strains have been designed so that even if they were to regain a single gene, they would still remain highly attenuated, and thus, they have been designated by many institutional biosafety committees to be safe to use under less restrictive BSL-2 conditions. But by design, none are virulent, and therefore, these strains are not useful in studies of pathogenesis.

**(iv) *Mycobacterium smegmatis*.** *M. smegmatis* has long been a favorite organism in many mycobacterial labs. It grows quickly and is remarkably safe. But its very advantage, its safety, is also a drawback. The common laboratory strain is not virulent in any host under any circumstances. *M. smegmatis* is quite useful for a range of mycobacterial studies, including cell biology and metabolism, but generalizing any results to *M. tuberculosis* must be done advisedly.

**(v) Other pathogenic mycobacteria.** Several other mycobacteria are animal and occasional human pathogens. While some of these grow fairly slowly (notably the cause of Johne’s disease, *Mycobacterium avium* subsp. *paratuberculosis*) or not at all *in vitro* (*Mycobacterium leprae*, the cause of human leprosy), others grow more rapidly than *M. tuberculosis* does (e.g., other *Mycobacterium avium* complex members) or even as rapidly as *M. smegmatis* does (e.g., *Mycobacterium marinum*, *Mycobacterium abscessus*). Because these bacteria are natural pathogens, they often have appropriate hosts for studies of pathogenesis. Many, however, differ from *M. tuberculosis* in critical ways. For example, *Mycobacterium ulcerans*, the cause of Buruli ulcer in humans, causes disease by releasing a lipid molecule (mycolactone) that causes tissue destruction (58). There does not appear to be a similar toxin that explains the virulence of *M. tuberculosis*.

**What do model hosts tell us about TB?**

*It depends on the question. Again*.

TB is a natural disease of humans. Although in some settings, humans can transmit to other animals (most notably primates and elephants), there is no zoonotic transmission of disease. So any model host is, by definition, a nonnatural host. Each model has its advantages and drawbacks (3).

**(i) Mice.** Mice have been the workhorses (or work-mice) for studying TB for decades. Mice can be experimentally infected via aerosol or intravenous routes and develop slowly progressive infection that eventually ends in death. Most disease is confined to the lung, but as in humans, infection does disseminate to other organs. The main advantages of the mouse model are the plentiful reagents for studying the murine immune response, relative low cost, ease of handling, and the ability to perform classical genetic studies.

Most mouse studies have been performed using a small number of inbred strains. In these mice, infection looks far different from infection of humans. For example, in humans, latency is the norm, but all mice develop progressive infection that proceeds more or less in synchrony. And the histopathology in these mice looks fairly different from humans, with no necrotizing granulomas.

Part of the problem might be the very advantage of these inbred animals—their lack of genetic diversity. In fact, loss of a single genetic locus results in animals that are both more susceptible to infection and have histopathologic responses that more closely resemble those of humans (59). Thus, there may be ways to make the mouse model more closely resemble human disease.

**(ii) Rabbits and guinea pigs.** Both of these animal models, rabbits and guinea pigs, have long been used to study TB. Both are susceptible, though guinea pigs are much more highly susceptible than either mice or rabbits. And, depending on the individual animal and the strain of *M. tuberculosis*, both form granulomas. These lesions often progress in a manner seen in humans. Compared to mice, though, both of these models are relatively expensive and lack most of the immunologic and genetic tools that are available for mice.

**(iii) Zebrafish.** Fish, like zebrafish, are naturally infected by the aquatic organism *Mycobacterium marinum*. While they are cold-blooded and very distantly related to the mammalian host that we care most about, they have some specific advantages that have proven very useful. Zebrafish genetics are well worked out, and producing hypomorphic fish is as easy as buying the appropriate nucleic acid morpholino. And the zebrafish embryo is transparent, allowing infections with fluorescently labeled bacteria to be monitored continuously in single animals (60, 61). These properties have permitted the study of the morphogenesis of host lesions in real-time and defined some of the characteristics of early infection that would otherwise be unobservable. And some of the genetic observations have yielded insights that have translated into human polymorphisms (62).

**(iv) Nonhuman primates.** The closest model to human disease uses our closest relatives. Both macaques and marmosets can be easily infected with *M. tuberculosis*. In macaques, approximately half of the animals develop progressive disease after bronchoscopic infection; the remainder are able to control infection (63, 64). Like humans, these animals develop a range of pathology. And this is an excellent model to study the interaction of TB with HIV or simian immunodeficiency virus (SIV) (65, 66).

However, nonhuman primate (NHP) experiments are exceedingly expensive, and there are a very limited number of facilities that have the capacity to perform them along with a very limited number of available animals. The immunologic reagents remain fairly limited (though improving). And genetics are near impossible (though marmosets, which are highly susceptible, are generally born as identical twins). Thus, NHP experiments are likely to be used primarily for validation, particularly for translational work that might make its way into humans.

**(v) Humans.** Given that there is no shortage of human disease, why use model hosts at all? There is the obvious drawback—it is difficult, often impossible, to do an interventional study safely and ethically. But the tools to study human infection continue to improve, at least allowing a deeper understanding of disease that can be correlated with animal and *in vitro* studies. Foremost among these tools is imaging. In particular, PET/CT can now be used to monitor the natural history of TB during and after treatment, and in ongoing work, in those with clinically apparent infection (4, 6). And better probes could allow imaging not only of host inflammatory cells but also of bacteria. Thus, it is likely that we will be able to obtain increasingly useful data from human studies.

**How does *M. tuberculosis* survive and grow during infection?**

*It takes a whole genome*.

The classic paradigm of bacterial pathogenesis revolves around regulation. When a pathogen is exposed to a host, it expresses a set of genes that encode specific virulence factors. These are often toxins or specialized secretory systems that allow the pathogen to modify the responses of host cells, either killing them or subverting their defenses. But this paradigm simply doesn’t work for *M. tuberculosis*. As an obligate pathogen, it never has to adapt to the host—it is always prepared for the host environment. Although *M. tuberculosis* has a large complement of regulators, unlike in many Gram-negative pathogens, there are no master regulators of virulence determinants.

Instead, the entire physiology of *M. tuberculosis* is geared for survival in the host. Because of this, it is quite difficult to define factors that are specifically designed for virulence; instead it has adapted factors it has inherited to take advantage of the host environment. This makes it difficult to define specific virulence factors, as mutations in a huge variety of metabolic pathways have deleterious effects during model infections. Certainly, *M. tuberculosis* does have factors that resemble more classic virulence determinants, most notably an alternative protein secretion system (ESX-1) whose loss is associated with decreased virulence (67, 68). But this locus is closely related to those found in other, nonpathogenic bacteria and, in fact, is one of several related ESX systems in *M. tuberculosis* that have unrelated functions. This is a consistent theme—rather than acquiring new exotic mechanisms of virulence, *M. tuberculosis* seems to adapt existing genetic loci to optimize survival within its host. For example, *M. tuberculosis* has utilized genes present in other relatives to develop an efficient cholesterol uptake and metabolism system, permitting it to use host-derived cholesterol as its major carbon source during infection (69, 70).

Most of what we know comes from model studies. In addition, there is information that can be derived from the natural variation in the *M. tuberculosis* population. In fact, the importance of ESX-1 was first recognized from studying BCG, whose attenuation is due, in part, to a large deletion in the ESX-1 locus. However, there is an obvious limitation to studying *M. tuberculosis* virulence using isolates from human disease. By definition, all of these are virulent, and nearly all are capable of being transmitted. Thus, we will probably always be limited to studying major determinants of virulence in experimental systems.

**How does the host respond?**

*It’s all hands on deck. But it’s hard to tell which hands are important*.

The numerous human host responses to *M. tuberculosis* infection have been extensively catalogued. Active TB induces a broad range of immune mechanisms, though these do vary considerably from individual to individual. There are CD4^+^ and CD8^+^ responses that can be measured in peripheral blood. In addition, recent evidence suggests that antibodies may play a role in human infection (71–73). But which of these is most important?

Most of what we know in human disease is derived from rare genetic variants and from acquired immunodeficiencies. For example, the importance of the IFN-γ–IL-12–IL-23 signaling pathway has been defined in individuals with unusual genetic diseases (14). The importance of tumor necrosis factor alpha (TNF-α) was actually recognized during the initial trials of antibodies that blocked the activity of this cytokine (74). In fact, treatment with anti-TNF-α agents remains a substantial risk factor for both the reactivation of latent TB and the development of primary progressive TB. And the unfortunate vast experience with HIV has demonstrated the importance of CD4^+^ T cells.

Which mechanisms are operative in controlling TB in individuals with normal immune systems? This remains a vexing question. To generalize, immune responses that are measurable in the peripheral blood are broadly similar in latent and active TB. Thus, it has been very difficult to find mechanisms that differentiate those who will develop clinical disease from those who will not. One possible reason for this might be connected with the observation that, while many lesions form in the infected lung, each lesion appears to progress independently of other lesions. This suggests that important immune phenomena are occurring at a local level, which might not be reflected in peripheral blood samples. In fact, considerable evidence from experimental animals suggests that local immune responses in the lung are far different from those seen in the periphery.

**What is a protective immune response to TB?**

*No idea*.

For many pathogens that cause acute disease, one infection provides protection against subsequent infection. It is less clear whether this is true for TB. Several older studies have shown that those with evidence of latent TB are less likely to develop active TB when exposed (75). However, it is not clear whether this indicates that they have been stimulated to have a protective immune response or that infection has defined them as the group that is constitutively less likely to develop disease upon exposure. And we do know that those who have been treated for TB can be reinfected and redevelop disease. Though, again, this might have little to do with immunity—their having had TB might mark them as a particularly susceptible group.

We are stuck in a bit of a Catch-22 (76). It is difficult to identify a protective response without an effective vaccine, but it is equally difficult to find a protective vaccine without a biomarker of protection. Unless there is a revelation from animal studies, knowing what protection looks like might require advances in vaccine development.

**Can we do better with TB diagnosis?**

*We’re already doing it*.

As discussed above, one of the largest advances in clinical TB has been the introduction of an automated system to diagnose TB using nucleic acid amplification (29). While it has always been clear that such a system could be more sensitive and specific than existing diagnostic approaches, success of this system is based on good engineering—the ability to achieve reproducible results without much human intervention. Moreover, the ability to detect drug resistance rapidly can truly transform the clinical care of patients. The system remains expensive (though heavily subsidized in much of the developing world), and further advances are certainly possible. For example, there are efforts under way to use the same or similar devices to diagnose extrapulmonary disease, a serious technical challenge, and to extend the ability to identify drug resistance beyond a single agent. But the success of this approach provides a template and a yardstick for further developments.

There have been two other promising approaches to improving diagnostics. One concentrates on detecting bacterial biomarkers in accessible compartments. The most advanced is detection of a bacterial glycolipid, lipoarabinomannan, in urine samples from patients using a simple antibody-based diagnostic similar to a home pregnancy test (77). Unfortunately, this seems to be fairly insensitive, though it might have a place in diagnosis in HIV patients (78). Other bacterial components might be detected in blood or as volatile compounds in exhaled air (79, 80). And while there are high-tech methods to identify volatile compounds, there has been some promise with using a decidedly low-tech approach—trained rats (81).

From the host side, it could be that a specific biomarker is generated by the host response to TB. Several efforts have been made to find transcriptional signatures of active TB in whole blood from both adults and children (82–84). These signatures seem to be able to differentiate between latent and active TB and, to various degrees, TB and other infections. This is still very much at a research stage and reducing the signatures to a clinically useful test may be challenging. But it is a challenge being taken on by several groups, and it continues to show promise.

**Can we do better with TB treatment?**

*Almost certainly*.

Current therapy suffers from two drawbacks. It takes a very long time, and the length of treatment adds to the expense and complexity of delivering care. And there is a tremendous need for more effective treatment of drug-resistant disease.

Shortening the course of therapy has been frustrating. It is clear from early studies that the majority of patients are cured far earlier than the recommended 6 months of treatment. However, without completing therapy, a significant minority will relapse. If we could identify the subgroup that is cured early, we could shorten treatment for most patients. Unfortunately, there are no biomarkers of cure. Thus, treatment is extended for everyone to prevent a small minority from relapsing.

Altering the treatment regimen is what led to the success of shorter therapeutic courses. Thus, perhaps, new drugs could permit further shortening. The first attempts to do that were not terribly successful. Substituting or adding fluoroquinolone drugs in standard treatment regimens failed to allow 4 months instead of 6 months of therapy in three large clinical trials (85–87). However, this concept remains promising. Studies of mice have shown that regimens that include newly introduced drugs can shorten therapy to as little as 6 weeks (88), though it will take human trials to determine the predictive power of the mouse model.

Several new drugs are either being developed or repurposed for TB. These drugs include antibiotics that are used in other infections, such as linezolid (89), and new classes of drugs, such as bedaquiline (90) and delamanid (91), with novel mechanisms of action. Each of these drugs has been shown to improve the response of drug-resistant TB, allowing shorter courses of treatment when added as single agents to otherwise poorly performing regimens (89, 92–94). Mouse studies have suggested that combining some of these antibiotics results in more rapid cure, a hypothesis that is currently being tested in humans.

**Can we do better with TB prevention?**

*Hopefully. But note the lack of confidence in that statement*.

The holy grail of clinical TB is a vaccine. A highly effective vaccine would likely be the single most cost-effective tool for controlling the TB epidemic. Modeling suggests that even a moderately effective vaccine could have a large impact.

The lack of efficacy of BCG in adult TB suggests several possible avenues for better vaccine development. What is wrong with BCG? Is it lacking critical antigens that are required for protection? If so, vaccines that include these antigens or live attenuated whole-cell vaccines that are more similar to *M. tuberculosis* might provide a better immune response. Or are the responses to antigens that exist in BCG inadequate to provide protection? If that is true, presenting vaccines in a novel manner might be more effective.

All of these strategies are being pursued. Several vaccine candidates, too many to cite here, have shown protection in mouse models that is better than that achieved with BCG. And some have had success in NHPs (95). How well these animal models predict human efficacy, however, is unclear. And human trials of TB vaccines are very large and expensive. One such trial, of a vaccinia virus-produced *M. tuberculosis* antigen given to infants after BCG vaccination, failed to show any efficacy (96). As a result, funders have, in general, taken a step back from such large clinical trials. Another large trial is likely to first require advances in understanding what protection looks like and in being able to generalize animal results to humans. At this point, it is not clear what those advances will look like.
